# Artificial Intelligence in Ophthalmology: A Comparative Analysis of GPT-3.5, GPT-4, and Human Expertise in Answering StatPearls Questions

**DOI:** 10.7759/cureus.40822

**Published:** 2023-06-22

**Authors:** Majid Moshirfar, Amal W Altaf, Isabella M Stoakes, Jared J Tuttle, Phillip C Hoopes

**Affiliations:** 1 Corneal and Refractive Surgery, Hoopes Vision Research Center, Draper, USA; 2 Ophthalmology, The University of Utah, Salt Lake City, USA; 3 Eye Banking and Corneal Transplantation, Utah Lions Eye Bank, Murray, USA; 4 Medical School, University of Arizona College of Medicine Phoenix, Phoenix, USA; 5 Medical School, Pacific Northwest University of Health Science, Yakima, USA; 6 Ophthalmology, Hoopes Vision Research Center, Draper, USA; 7 Medical School, University of Texas Health Science Center at San Antonio, San Antonio, USA

**Keywords:** cornea, chatgpt-4, chatgpt-3.5, conversational generative pre-trained transformer, chatbot, ophthalmology, clinical decision-making, conversational ai, statpearls, artificial intelligence

## Abstract

Importance

Chat Generative Pre-Trained Transformer (ChatGPT) has shown promising performance in various fields, including medicine, business, and law, but its accuracy in specialty-specific medical questions, particularly in ophthalmology, is still uncertain.

Purpose

This study evaluates the performance of two ChatGPT models (GPT-3.5 and GPT-4) and human professionals in answering ophthalmology questions from the StatPearls question bank, assessing their outcomes, and providing insights into the integration of artificial intelligence (AI) technology in ophthalmology.

Methods

ChatGPT's performance was evaluated using 467 ophthalmology questions from the StatPearls question bank. These questions were stratified into 11 subcategories, four difficulty levels, and three generalized anatomical categories. The answer accuracy of GPT-3.5, GPT-4, and human participants was assessed. Statistical analysis was conducted via the Kolmogorov-Smirnov test for normality, one-way analysis of variance (ANOVA) for the statistical significance of GPT-3 versus GPT-4 versus human performance, and repeated unpaired two-sample t-tests to compare the means of two groups.

Results

GPT-4 outperformed both GPT-3.5 and human professionals on ophthalmology StatPearls questions, except in the "Lens and Cataract" category. The performance differences were statistically significant overall, with GPT-4 achieving higher accuracy (73.2%) compared to GPT-3.5 (55.5%, p-value < 0.001) and humans (58.3%, p-value < 0.001). There were variations in performance across difficulty levels (rated one to four), but GPT-4 consistently performed better than both GPT-3.5 and humans on level-two, -three, and -four questions. On questions of level-four difficulty, human performance significantly exceeded that of GPT-3.5 (p = 0.008).

Conclusion

The study's findings demonstrate GPT-4's significant performance improvements over GPT-3.5 and human professionals on StatPearls ophthalmology questions. Our results highlight the potential of advanced conversational AI systems to be utilized as important tools in the education and practice of medicine.

## Introduction

Chat Generative Pre-Trained Transformer (ChatGPT) is a natural language processing model that was developed by OpenAI and released in November 2022 [1]. It has gained significant recognition in the healthcare field, particularly due to its noteworthy performance in passing all three parts of the United States Medical Licensing Exam (USMLE), achieving scores near or at 60% in each examination [2]. In addition to its success in answering a majority of medicine board questions, ChatGPT has also demonstrated competence in graduate-level business and law school examinations, achieving scores of B and C+, respectively [3,4].

In the field of ophthalmology, artificial intelligence (AI) models like ChatGPT can play a crucial role in answering patient-specific management questions, providing quick insights on relevant criteria, and facilitating discussions on ocular conditions, treatments, and procedures. However, despite its success in the overall fields of medicine, business, and law, ChatGPT has been shown to have variable accuracy in answering specialty-specific medical questions [5,6].

OpenAI has developed two prominent iterations of the ChatGPT model, namely GPT-3.5 and GPT-4. The latest version, GPT-4, boasts improved efficiency and accuracy compared to its predecessor. Notable advancements include enhanced contextual understanding, improved language fluency, and an expanded knowledge base [7]. However, it remains uncertain whether GPT-4 possesses an enhanced ability to comprehend nuanced medical terminology and provide more accurate and contextually relevant responses, particularly within the domain of ophthalmology.

This study aims to evaluate the performance of GPT-4, GPT-3.5, and human professionals in answering ophthalmology questions sourced from the StatPearls question bank, a resource that provides practice questions commonly used by residents, fellows, and attendings to prepare for board exams and clinical practice [8]. The primary objective is to assess their question-answer percent correct outcomes, while the secondary objective involves stratifying the results by difficulty level and ophthalmology subcategories. By examining the strengths and limitations of GPT-3.5 and GPT-4 in addressing higher-level ophthalmology questions, this study aims to contribute to the ongoing dialogue surrounding the integration of AI technology in healthcare, ultimately striving to improve patient care within the field of ophthalmology.

## Materials and methods

ChatGPT's performance was evaluated utilizing StatPearls ophthalmology questions, which are peer-reviewed questions from a medical question bank designed to be at the level of ophthalmology residents, fellows, and attendings. Free StatPearls questions were accessed via the National Library of Medicine bookshelf (https://www.ncbi.nlm.nih.gov/books/NBK430685/). “Ophthalmology” was used as a search criterion to filter relevant StatPearls questions. The search yielded 548 entries or 1,096 questions. The results were sorted in alphabetical order and a random number generator was used to select each entry. The question was omitted if it was not directly relevant to ophthalmology. The questions were then pasted into ChatGPT, preceded by the statement, “Please choose the best answer and explain your reasoning:”

GPT-3.5 and 4 were given the same 467 questions between May 9, 2023, and May 26, 2023. A new chat session was used for each question to avoid memory retention bias. Questions with images in the prompt were included with the accessibility verbal image description produced from copying and pasting it so long as these descriptions did not nullify the question. ChatGPT was able to refer to these image descriptions in its responses, indicating its ability to synthesize image information despite not being able to process images.

To assess human performance in answering these questions, the data from the StatPearls website for each question were recorded. Question difficulty on a scale of 1 to 4 was also recorded, based solely on StatPearls difficulty grading. Level 1 indicated a “basic” difficulty level and tested recall; Level 2 indicated “moderate” difficulty and tested the ability to comprehend basic facts; Level 3 was described as “difficult” and tested application, or knowledge use in care; Level 4 was considered an “expert” high complexity question and tested analysis and evaluation skills. While StatPearls does not provide specific data regarding the precise count of human learners who responded to individual questions, we can say there was at least one human respondent for each question and the average percent correct of human responses within a given category was calculated.

The 2017 Ophthalmic Knowledge Assessment Program (OKAP) Exam Content Outline was referenced when creating ophthalmology subcategories in which to organize the questions [9]. The questions were sorted into the following American Association of Ophthalmology (AAO)-defined subcategories: clinical optics; ophthalmic pathology and intraocular tumors; neuro-ophthalmology; pediatric ophthalmology and strabismus; oculoplastics; external disease and cornea; intraocular inflammation and uveitis; glaucoma; lens and cataract; retina and vitreous; and refractive surgery. The categories were further grouped as follows: cornea, cataracts, and refractive surgery comprising the “anterior segment;” retina comprising the “posterior segment;” and neuro-ophthalmology, pediatrics, and oculoplastics comprising the “other” category. Questions from the glaucoma, pathology, and uveitis categories were individually divided amongst the “anterior,” “posterior,” and “other” categories according to question content.

ChatGPT responses were organized by GPT model, question difficulty on a scale of 1 to 4, ophthalmology subcategory, and generalized anatomical category. Performance of GPT-3.5, GPT-4, and humans in answering ophthalmology practice questions was measured as the proportion of correct answers. Our secondary outcomes were the proportion of questions GPT-3.5, GPT-4, and humans were able to answer correctly within different difficulty levels.

The study demonstrated a power of 0.80. Each of the three groups underwent a set of 467 questions, resulting in a sample size of 1,401. The effect size of 0.083, calculated using the G*Power software (Version 3.1.9.6, Düsseldorf, Germany), was employed as a parameter in the analysis.

Statistical analysis was performed utilizing Microsoft Excel (Version 16.73, Redmond, WA) and SPSS Statistics Software (IBM Corp. Released 2022. IBM SPSS Statistics for Windows, Version 29.0. Armonk, NY: IBM Corp). The Kolmogorov-Smirnov test was used to test for normality. Due to the normal distribution of the data, a one-way analysis of variance (ANOVA) was used to test for the statistical significance of GPT-3 versus GPT-4 versus human performance. To compare the means of the two groups, repeated unpaired two-sample t-tests were used. P-values were 2-tailed where applicable, and a p-value of less than 0.05 was considered statistically significant.

## Results

**Figure 1:**
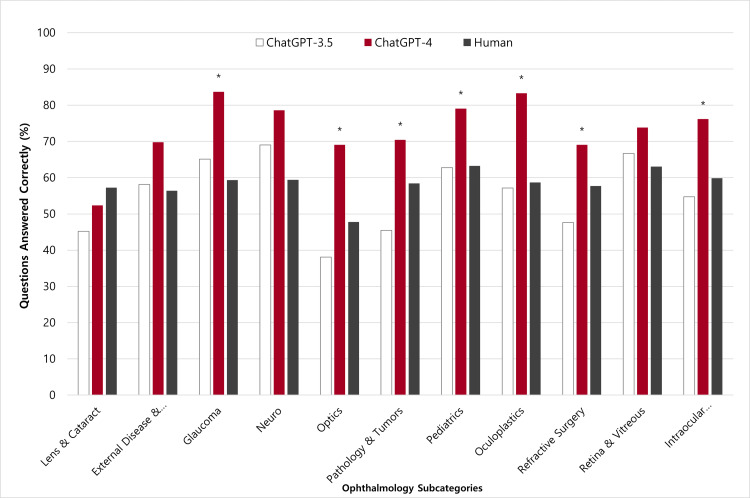
Comparing the performance of GPT-3.5, GPT-4, and human professionals on StatPearls questions divided into ophthalmology sub-categories * indicates statistical significance

GPT-4 performed significantly better than both GPT-3.5 (73.2% vs. 55.46%; p < 0.001) and humans (73.23% vs. 58.31%, p < 0.001) on 467 ophthalmology StatPearls questions. When comparing individual categories, GPT-4 performed superior to both GPT-3.5 and humans in all categories except “lens and cataract”, in which humans performed better (Figure 1).

**Figure 2:**
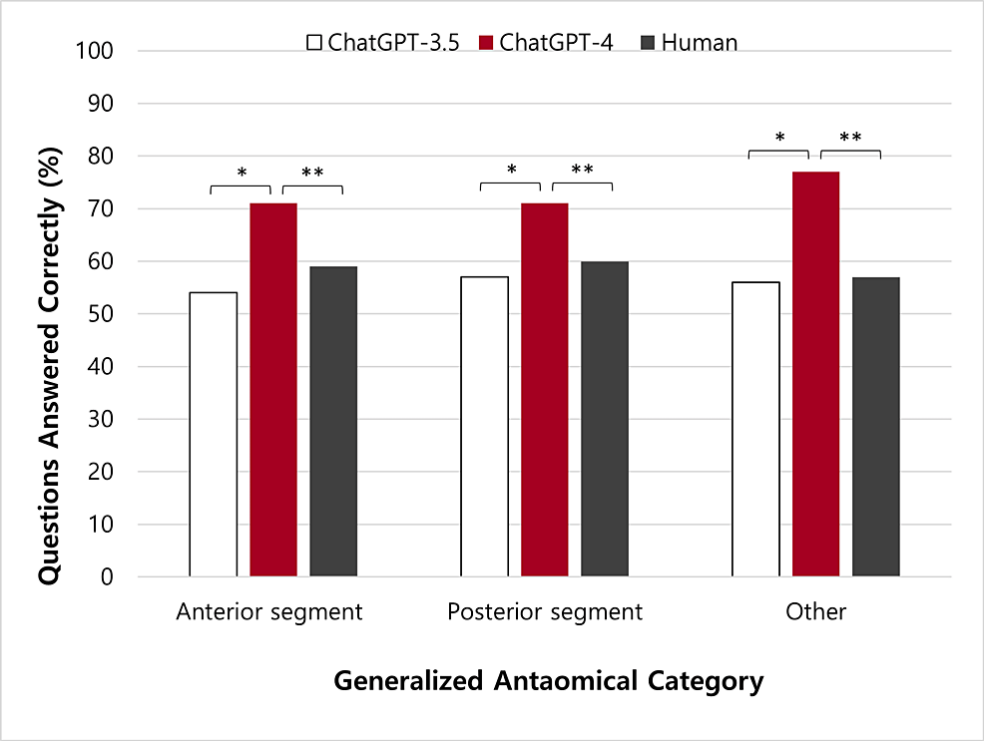
Comparing the performance of GPT-3.5, GPT-4, and humans on StatPearls questions divided into generalized anatomically based categories The “anterior segment” included cornea, cataract, and refractive surgery categories; the “posterior segment” included the retina and vitreous category; the “other” category was comprised of neuro-ophthalmology, pediatrics, and oculoplastics. Questions from the glaucoma, pathology, and uveitis categories were individually divided amongst the “anterior,” “posterior,” and “other” categories according to question content. *, ** indicates statistical significance

Although GPT-4 demonstrated improved performance over GPT-3.5 and human professionals in ten categories, the differences were not statistically significant in the domains of “external disease and cornea,” “neuro-ophthalmology,” and “retina and vitreous.” The percentage of questions answered correctly was also found for the generalized anatomical categories (Figure 2, Table 1).

**Table 1:** Percentage of questions answered correctly by GPT-3.5 vs. GPT-4 vs. humans by ophthalmology sub-category Bolding indicates statistical significance

Ophthalmology Subcategory	GPT-3.5 Questions Answered Correctly (%)	GPT-4 Questions Answered Correctly (%)	Human Questions Answered Correctly (%)	GPT-3.5 vs GPT-4 P-Value	GPT-3.5 vs Human P-Value	GPT-4 vs Human P-Value
Lens & Cataract (n = 42)	45	52	57	0.518	0.163	0.569
External Disease & Cornea (n = 43)	58	70	56	0.267	0.833	0.085
Glaucoma (n = 43)	65	84	59	0.048	0.614	0.003
Neuro (n = 42)	69	79	59	0.327	0.212	0.007
Optics (n = 42)	38	69	48	0.004	0.284	0.017
Pathology & Tumors (n = 44)	45	70	58	0.017	0.124	0.128
Pediatrics (n = 43)	63	79	63	0.099	0.951	0.023
Oculoplastics (n = 42)	57	83	59	0.008	0.851	< 0.001
Refractive Surgery (n = 42)	48	69	58	0.002	0.397	0.003
Retina & Vitreous (n = 42)	67	74	63	0.480	0.652	0.157
Intraocular Inflammation & Uveitis (n = 42)	55	76	61	0.039	0.496	0.054
Total (n = 467)	55	73	58	< 0.001	0.231	<0.001

**Figure 3:**
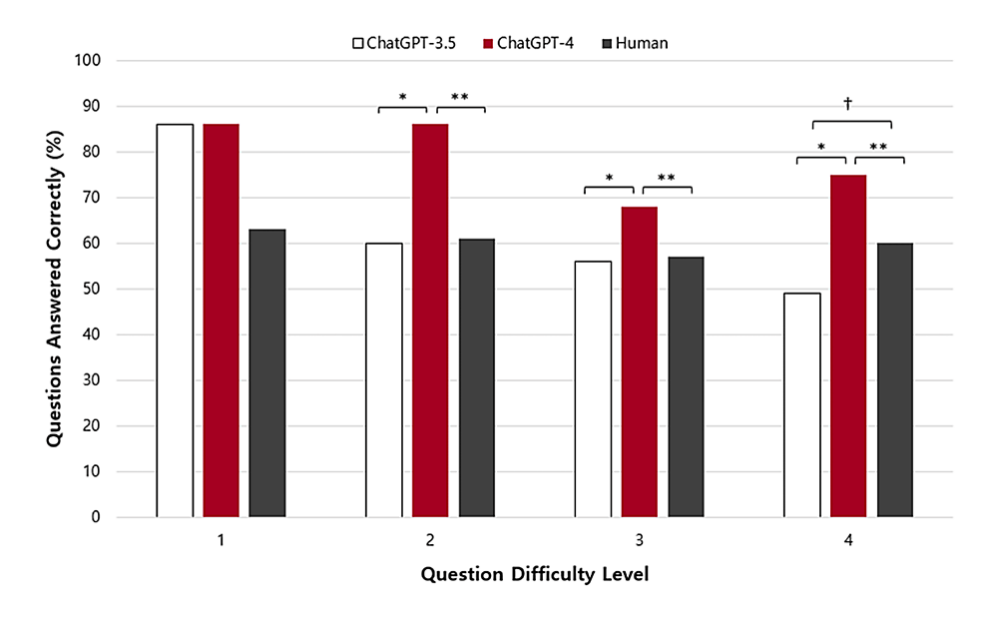
Comparing the performance of GPT-3.5, GPT-4, and humans on StatPearls questions divided by difficulty levels Level 1 indicated the “basic” difficulty level and tested recall; Level 2 indicated “moderate” difficulty and tested the ability to comprehend basic facts; Level 3 was described as “difficult” and tested application, or knowledge use in care; Level 4 was considered an “expert” high-complexity question and tested analysis and evaluation skills. *, **, † indicates statistical significance

When sorted by difficulty level, there were significant differences in the performance of the three models within difficulty levels two, three, and four (Figure 3, Table 2).

**Table 2:** Percentage of questions answered correctly by GPT-3.5 vs. GPT-4 vs. humans by generalized anatomical category and difficulty level The “anterior segment” included the cornea, cataract, and refractive surgery categories; the “posterior segment” included the retina and vitreous category; the “other” category consisted of neuro-ophthalmology, pediatrics, and oculoplastics. Questions from the glaucoma, pathology, and uveitis categories were individually divided amongst the “anterior,” “posterior,” and “other” categories according to question content. Level 1 indicated the “basic” difficulty level and tested recall; Level 2 indicated “moderate” difficulty and tested the ability to comprehend basic facts; Level 3 was described as “difficult” and tested application, or knowledge use in care; Level 4 was considered an “expert” high-complexity question and tested analysis and evaluation skills. Bolding indicates statistical significance.

		GPT-3.5 Questions Answered Correctly (%)	GPT-4 Questions Answered Correctly (%)	Human Questions Answered Correctly (%)	GPT-3.5 vs GPT-4 P-Value	GPT-3.5 vs Human P-Value	GPT-4 vs Human P-Value
Question Difficulty Level	1 (n = 7)	86	86	63	1	0.176	0.176
	2 (n = 278)	60	86	61	<0.001	0.513	<0.001
	3 (n = 265)	56	68	57	<0.001	0.662	<0.001
	4 (n = 117)	49	75	60	<0.001	0.008	<0.001
Generalized Anatomical Category	Anterior segment (n = 208)	54	71	59	< 0.001	0.211	< 0.001
	Posterior segment (n = 75)	57	71	60	0.033	0.657	0.034
	Other (n = 184)	56	77	57	< 0.001	0.725	< 0.001

GPT-3.5 and GPT-4 performed similarly on level-one questions, with both versions outperforming human professionals (86% vs. 63%, respectively). However, this was not a statistically significant difference, likely due to the small sample size of level-one questions (n = 7). GPT-3.5 and human professionals performed similarly on level two and three questions while humans significantly outperformed GPT-3.5 on level-four questions (49% vs. 60%, p < 0.001). GPT-4 performed superior to both GPT-3.5 and humans on level two, three, and four questions (p < 0.001). Despite the variability in performance exhibited by GPT-3.5 and GPT-4 across difficulty levels, human performance remained relatively consistent. Acknowledging that GPT is incapable of directly analyzing images referenced within questions, the inclusion of images in the question stem did not yield any statistically significant differences in the performance of GPT-3.5 and GPT-4.

## Discussion

Advancements in artificial intelligence have had profound impacts on many industries, including healthcare. One significant application of AI in healthcare is the development of conversational AI models, such as ChatGPT, which have the potential to assist medical professionals in providing accurate and timely information.

Recent studies have focused on examining ChatGPT's roles in clinical decision-making, generating differential diagnoses, and providing insights into treatment options [10-12]. In addition to these roles, ChatGPT can serve as a valuable tool for medical education, helping healthcare professionals stay updated with the latest advancements and improve their overall clinical practice. However, despite the progress ChatGPT has made since its inception, its accuracy in the application of medical specialty and subspecialty knowledge is still under investigation. In a study conducted by Mihalache et al., the performance of ChatGPT in answering ophthalmology questions was investigated using OphthoQuestions, a popular question bank for the OKAP and Written Qualifying Exam (WQE) taken by ophthalmology residents [13]. Over the course of just one month (January 2023 - February 2023), ChatGPT's accuracy in providing correct answers increased from 46% to 58%. However, the authors noted that this improvement was insufficient to establish ChatGPT as a reliable study tool for the OKAP/WQE. This study offers valuable insights into ChatGPT's capabilities in the field of ophthalmology in addition to our study comparing ophthalmology-related performance not only between GPT-3.5 and GPT-4 versions but also comparing their performance and that of humans. The chatbot's varying accuracy in specialized knowledge is further illustrated by its passing performance in the European Exam in Core Cardiology and the Plastic Surgery In-Service Exam, juxtaposed with its inadequacy in the American Board of Orthopaedic Surgery Examination and the American College of Gastroenterology Self-Assessment Test [5,6,14,15]. In view of this, the results of this study indicate a substantial improvement in the performance of GPT-4 compared to its predecessor and human participants.

When evaluating the performance of the models in individual subspecialty categories, GPT-4 demonstrated superiority over both GPT-3.5 and humans in all categories except for the "lens and cataract" category. While this category did not contain an increased number of higher-order questions compared to the other categories, it is possible it may have posed specific challenges that affected the performance of GPT-4. Such difficulties could stem from the presence of specific enhancements in the field of lens and cataract that may not be updated or reflected in ChatGPT’s training. Alternatively, it is conceivable that ophthalmology residents and fellows are more familiar with cataracts and lens-related topics, as they constitute the fundamentals of clinical practice. Further investigation into the specific difficulties encountered within this category could provide valuable insights for future model enhancements.

The differences in performance between the models and humans varied across difficulty levels (Figure 3). When the questions were sorted by difficulty, significant differences were observed between the three models within difficulty levels 2, 3, and 4 (Table 2). The varying performance of GPT-3.5 and GPT-4 across difficulty levels suggests the presence of nuanced challenges that affect their capabilities in comprehending and responding to different levels of complexity, particularly for GPT-3.5, whose performance declined with each increase in difficulty level. However, images in the question stem did not yield any statistical differences, and we theorize that this is due to both GPT-3.5 and GPT-4's ability to use the written accessibility descriptions adequately. In contrast, human performance remained relatively consistent across higher and lower-order difficulty levels, perhaps highlighting the adaptability of human cognitive abilities in handling varying levels of difficulty in comparison to the AI-based models. Though not statistically significant due to the small sample size of basic level-1 questions, AI models performed notably better than humans on these recall-based questions, likely due to the ability of the models to draw from an accessible and established knowledge base.

The findings of this study have significant implications for the field of ophthalmology and the development of conversational AI systems. The superior performance of GPT-4 over both GPT-3.5 and humans suggests substantial rapid advancements in AI-based question-answering systems. GPT-4, released in March 2023, was able to achieve or surpass human performance within four months of its predecessor's release in November 2022. Earlier studies have highlighted the performance distinction between GPT-4 and GPT-3.5 on the OKAP exam with inferior accuracy of GPT-3.5 [16,17]. Similar findings were also observed when testing the difference between the two models on the dermatology SCE examination [18]. Our investigation, encompassing a significant volume of ophthalmology questions, aligns with and reinforces this observed variance in performance.

These improvements may enhance the accessibility and accuracy of medical information for healthcare professionals and patients alike, thereby potentially improving patient care and outcomes. However, it is important to note that GPT-4 is currently less accessible than GPT-3.5. Members must pay a monthly fee to use GPT-4 and are limited to 25 messages over the course of three hours. Additionally, both GPT-3.5 and GPT-4 have a knowledge cutoff of September 2021, which may limit the accuracy of these models in providing responses within a field that is constantly evolving. At present, these factors may be considered a barrier to the use of ChatGPT in clinical practice and education, as GPT-3.5 was shown to have inferior reliability in answering questions of varying difficulty and ophthalmic content.

The current study has some limitations. First, it cannot be conclusively stated that these questions are only answered by ophthalmology residents, fellows, and attendings, though there are many factors that contribute to the answering pool consisting of only these groups such as a financial subscription and inputting proper credentials into the StatPearls portal for the respective question banks. Second, the evaluation was focused solely on ophthalmology StatPearls questions, which may not fully capture the range of complexities encountered in clinical practice. Although StatPearls has the credibility for the education of residents and fellows in various medical specialties, better standardization may be necessary to differentiate clinical judgment from mere factual knowledge. It cannot be conclusively stated that StatPearls multiple-choice questions are a gold standard for demonstrating enhanced cognitive understanding of medical thought processes by AI. Further studies should include a broader set of specialty-specific medical questions to assess the generalizability of the observed performance differences. Additionally, the human participants in this study may not represent the entire population of healthcare professionals, and their performance may be influenced by individual variations in expertise and experience.

## Conclusions

The results of this study demonstrate the significant improvements in performance achieved by GPT-4 compared to GPT-3.5 and human professionals on ophthalmology questions sourced from a professional-level question bank. While GPT-4 outperformed both GPT-3.5 and humans in most categories and difficulty levels, the “lens and cataract” category presented a unique challenge for the model. These findings highlight the potential of advanced conversational AI systems in the medical domain and emphasize the need for further research and development to address specific challenges encountered in different medical specialties.

We support the use of AI as an adjunct in education and medicine rather than a replacement for human professionals. The results of this study indicate the advanced ability of AI models to answer multiple-choice questions, but the model’s performance on these questions is not a direct indicator of clinical aptitude. While the rapid advancement of GPT-4’s ophthalmic question-answering ability may foreshadow an increasing incorporation of the AI model into medical practice, an emphasis should be placed on using AI systems both responsibly and cautiously.
